# A novel hypoxia-associated gene signature for prognosis prediction in head and neck squamous cell carcinoma

**DOI:** 10.1186/s12903-023-03489-8

**Published:** 2023-11-14

**Authors:** Jingyi Luo, Yuejiao Huang, Jiahe Wu, Lin Dai, Mingyou Dong, Bo Cheng

**Affiliations:** 1 Department of Stomatology, Zhongnan Hospital of Wuhan University, No. 169 Donghu Road, Wuchang District, Wuhan, 430071 China; 2 School of Laboratory Medicine, Youjiang Medical College for Nationalities, No. 98 Chengxiang Road, Youjiang District, Baise, 533000 China; 3 Department of Cardiology, Zhongnan Hospital of Wuhan University, Wuhan, China; 4 Department of Stomatology, Wuhan No. 1 Hospital, No. 215 Zhongshan Road, Qiaokou District, Wuhan, 430030 China

**Keywords:** Hypoxia, HNSCC, TCGA, Prognostic, Biomarker

## Abstract

**Background:**

Head and neck squamous cell carcinoma (HNSCC) is the most common malignant tumor of head and neck, which seriously threatens human life and health. However, the mechanism of hypoxia-associated genes (HAGs) in HNSCC remains unelucidated. This study aims to establish a hypoxia-associated gene signature and the nomogram for predicting the prognosis of patients with HNSCC.

**Methods:**

Previous literature reports provided a list of HAGs. The TCGA database provided genetic and clinical information on HNSCC patients. First, a hypoxia-associated gene risk model was constructed for predicting overall survival (OS) in HNSCC patients and externally validated in four GEO datasets (GSE27020, GSE41613, GSE42743, and GSE117973). Then, immune status and metabolic pathways were analyzed. A nomogram was constructed and assessed the predictive value. Finally, experimental validation of the core genes was performed by qRT-PCR.

**Results:**

A HNSCC prognostic model was constructed based on 8 HAGs. This risk model was validated in four external datasets and exhibited high predictive value in various clinical subgroups. Significant differences in immune cell infiltration levels and metabolic pathways were found between high and low risk subgroups. The nomogram was highly accurate for predicting OS in HNSCC patients.

**Conclusions:**

The 8 hypoxia-associated gene signature can serve as novel independent prognostic indicators in HNSCC patients. The nomogram combining the risk score and clinical stage enhanced predictive performance in predicting OS compared to the risk model and clinical characteristics alone.

**Supplementary Information:**

The online version contains supplementary material available at 10.1186/s12903-023-03489-8.

## Introduction

Head and neck squamous cell carcinoma (HNSCC) is the most common malignancy of the head and neck, arising from the mucosal epithelium of the mouth, pharynx, and larynx. Each year, HNSCC is identified in over 870,000 new cases worldwide, killing about 440,000 people [1]. Smoking, alcohol consumption, Human Papilloma Virus (HPV) infection and exposure to environmental pollutants are all risk factors for HNSCC [2, 3]. Although HNSCC can be treated with surgical resection supplemented with radiotherapy or chemotherapy plus radiotherapy, HNSCC patients’ 5-year survival rate keeps low since there are few early diagnoses [4]. Therefore, to develop new therapies and improve patient prognoses, it is essential to identify new predictive biomarkers for HNSCC.

Over the past few years, high-throughput sequencing and RNA sequencing have made it easier to develop molecular markers, which have made individualized treatment and better cancer prognosis possible [5]. Among these, the presence of hypoxia in solid tumors is an intrinsic characteristic and the role of HAGs in cancer is receiving increasing attention [6]. In addition, the role of hypoxia in tumor angiogenesis, cell proliferation, differentiation, and apoptosis has been established [7, 8]. Hypoxia influences the immune microenvironment and is linked to a poor patient prognosis [9]. In HNSCC, hypoxia induces epithelial-mesenchymal transition (EMT) which provides a powerful driver for tumor progression [10] and enhances the proliferation, migration, and invasion of tumors [11, 12]. Ding et al. reported immune cells infiltrated into HNSCC in a variety of risk groups [13].

In this study, public databases were employed to evaluate the mRNA profiles and associated clinical characteristics of HNSCC patients. Univariate and multivariate Cox regression algorithms were used to screen the HAGs related to the prognosis of HNSCC from The Cancer Genome Atlas (TCGA) database. Prognostic features were then established in TCGA database and validated using Gene Expression Omnibus (GEO) datasets. Afterward, we explored potential mechanisms of prognosis by exploring the relationship between the risk model and immune status. Finally, the expression levels of core genes in HNSCC were validated by quantitative real-time polymerase chain reaction (qRT-PCR).

## Methods

### Data sources

The raw RNA sequence (RNA-seq) data and corresponding clinical parameters of 502 HNSCC patients and 44 adjacent normal tissues were obtained from the TCGA database on 23 May 2022. A total of 499 HNSCC patients with complete clinical information and survival data were included for further analysis. For external validation purposes, RNA-seq data and clinical parameters of HNSCC patients were obtained from HNSCC-related mRNA datasets (GSE27020, GSE41613, GSE42743, and GSE117973) in the GEO database. The clinical baseline data of all included patients are shown in Additional file 1: Table S1 (Patient baseline information table) and Additional file 2 (Details of patients in the TCGA database).

### Identification and functional enrichment analysis of differentially expressed genes associated with hypoxia

A total of 200 HAGs were downloaded from the Molecular Signatures Database (https://www.gsea-msigdb.org/gsea/msigdb/cards/HALLMARK_HYPOXIA.html). The same approach was used to obtain HAGs in the previously published study [14]. The "limma" package of R software was performed to distinguish the differentially expressed HAGs between HNSCC and adjacent normal tissues [15]. The FDR was adjusted by the Benjamini–Hochberg method. FDR < 0.05 and |logFC|> 1.0 was set as the cut-off criteria of differently expressed HAGs. To explore the biological functions of the hypoxia-associated gene signature, GO and KEGG enrichment analyses were performed by the R-package "Clusterprofiler" (version 4.0) [16, 17].

### Development and validation of prognostic features

To establish a prognostic model, the R package "glmnet" was used to perform the univariate Cox regression and LASSO analyses on the differentially expressed genes screened previously. The penalty factor λ was identified by the minimum parameters. Next, a risk prognostic model was developed by multi-factor Cox regression, and the following formula was used to calculate the risk score: Risk Score = Gene1 CoefixExpi + Gene2 CoefixExpi + …GeneN CoefixExpi (Coef: coefficients, Exp: gene expression levels). 499 HNSCC patients from the TCGA database were classified into low and high risk subgroups based on the median risk score. Subsequently, the overall survival curves of different subgroups were compared by Kaplan–Meier analysis, the overall survival at 1, 3, and 5 years was described by time-dependent receiver operating characteristic (ROC) analysis, and the area under the curve (AUC) was used to access the model’s predictive power. Finally, this prognostic gene signature was also demonstrated to have prognostic value in predicting OS in HNSCC patients using the datasets GSE27020, GSE41613, GSE42743, and GSE117973.

### Prognostic value of the 8-gene prognostic model independent of other clinical characteristics

The TCGA-HNSCC samples were randomized into two groups to clarify the association between the prognostic model and various clinical characteristics, such as staging, grade, age, gender, T stage, N stage, and M stage. There were two subgroups of patients: stage I/II and III/IV subgroups, grade I/II and III/IV subgroups, age < 60 and age ≥ 60 subgroups, male and female subgroups, T0-T2 and T3/4 subgroups, N0 and N + subgroups, and M0 and M1 + Mx subgroups, respectively. To confirm the 8-gene prognostic features’ independent prognostic value, Kaplan–Meier survival analysis was performed on specific subgroups with various clinical characteristics.

### Analysis of immune infiltration

Immune differences between the two groups of the TCGA database were synthesized by using computational methods for assessing immune infiltration and function, including ESTIMATE [18], TIMER [19], MCP-counter [20], CIBERSORTx [21], and single-sample gene set enrichment analysis (ssGSEA). A two-sample Wilcoxon test was applied to compare immune infiltration and immune-related functions between the high and low risk groups.

### Construction and evaluation of the nomogram

Based on the TCGA HNSCC cohort, a nomogram containing characteristic risk scores and other clinical characteristics was developed to better predict HNSCC prognosis. Using univariate Cox regression analysis, clinical characteristics with significant associations with HNSCC prognosis were screened. Then, the variables screened in the previous step were included in a multivariate Cox regression analysis to search for independent predictive variables for OS in HNSCC patients and to construct the nomogram by statistically significant variables. Finally, the predictive performance of the established nomogram, risk score, and clinical characteristics was compared by using the decision curve analysis (DCA), calibration curves, ROC curves, and consistency index (C-index).

### Identification of risk score-related genes and functional enrichment analysis

To better understand the biological processes of hypoxia-associated genes, the most relevant genes (Pearson |R|> 0.5, *P* < 0.05) were identified in the TCGA database, and functional enrichment analysis was performed using the R-package "Clusterprofiler". Subsequently, gene set variation analysis (GSVA) enrichment analysis [22] and Gene set enrichment analysis (GSEA) [23] were used to screen the HNSCC cohort from TCGA for signaling pathways significantly associated with risk groupings and to screen out significant pathways according to a false discovery rate (FDR) < 0.05. Benjamini–Hochberg method was used to correct the FDR.

### Validation by the quantitative real-time polymerase chain reaction

The Zhongnan Hospital of Wuhan University provided nine pairs of HNSCC and adjacent non-cancerous tissues for this study, all of which were authorized by the ethics committee. Surgically resected patients with HNSCC who did not receive chemotherapy or radiotherapy were recruited for the study. Additional file 1: Table S2 presents the baseline information of the included clinical samples. Total RNA was extracted from tissues using TriQuick Reagent (Solarbio, Beijing, China, R1100); reverse transcription was carried out using a Prime Script RT kit (TaKaRa, Dalian, China, RR037A); and quantitative PCR was performed using standard protocols from the SYBR Green PCR kit (Toyobo, Osaka, Japan, QPK-201). The primer sequences for PCR are shown in Additional file 1: Table S3. Candidate genes’ relative mRNA levels were normalized to GAPDH mRNA expression, while differences were compared using paired t-tests. The changes were calculated using the 2^−∆∆Ct^ method. Data were presented as the mean values ± standard error of the mean (SEM) from at least three independent experiments. To further verify the differences in eight core genes between high and low risk groups for HNSCC. Tumor samples from 16 HNSCC patients were relatively quantified using qRT-PCR. The relative expression levels of 8 genes were input into the risk model and the risk score was calculated. The patients were divided into high and low risk groups according to the median risk score. qRT-PCR results were used to further compare the gene expression differences between high and low risk groups.

### Statistical analysis

The Student’s t-test and one-way ANOVA were applied to compare continuous variables, while the chi-square test was used to compare categorical variables. Using a log-rank test, Kaplan–Meier survival curves were compared. Hazard ratios (HRs) and 95% confidence intervals (CIs) for genes and clinical parameters associated with hypoxia were calculated using univariate and multivariate Cox regressions. Statistical analyses were performed using R software (v4.0.2). *P* < 0.05 was considered a significant level.

## Results

### Identification of differential hypoxia-associated genes in HNSCC

**Fig. 1:**
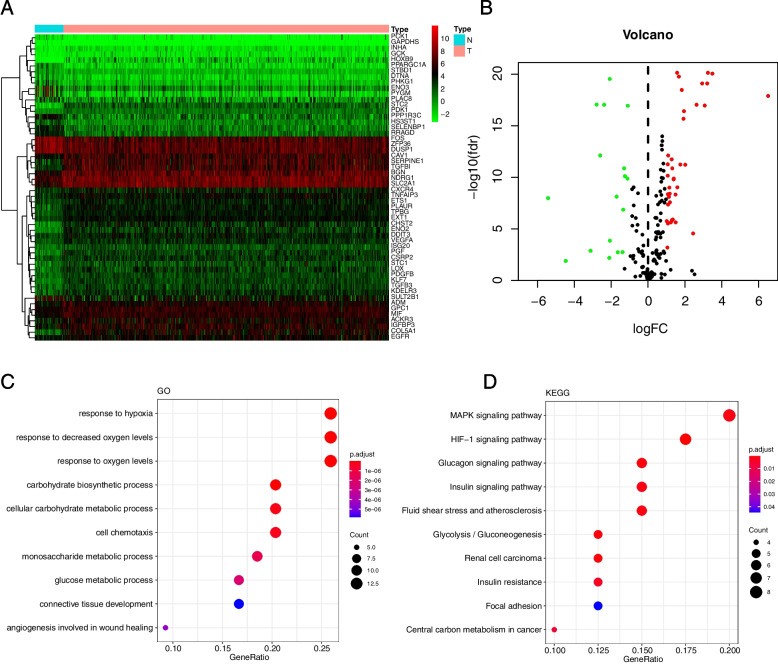
Identification of differentially expressed hypoxia-associated genes in HNSCC. **A** Heatmap of differentially expressed hypoxia-associated genes. N, Normal control group; T, HNSCC tumor group. **B** Volcano plot of differentially expressed hypoxia-associated genes. The abscissa represents the number of differentially expressed folds, and the ordinate represents the magnitude of the corrected FDR. **C** The bubble map shows the GO annotation of differentially expressed hypoxia-associated genes. **D** The bubble map shows the KEGG enrichment analysis of differentially expressed hypoxia-associated genes

First, differential expression analysis of 200 HAGs was performed to identify differentially expressed HAGs between HNSCC (*n* = 502) and normal samples (*n* = 44). The results showed that the RNA expression of 54 HAGs was significantly different, which are presented as heatmap and volcano plot (adjusted *p* < 0.05, Fig. 1A-B). Then, GO enrichment analysis was performed on these 54 HAGs, which identified the main pathways involved in these 54 genes: response to hypoxia/response to oxygen levels/cell chemotaxis/glucose metabolic process (Fig. 1C). Finally, the KEGG enrichment analysis was also performed on these 54 HAGs, and the findings revealed that they were involved in the following signaling pathways: MAPK signaling pathway, HIF-1 signaling pathway, insulin signaling pathway, and focal adhesion (Fig. 1D).

### Development of prognostic biomarkers for hypoxia-associated genes

**Fig. 2:**
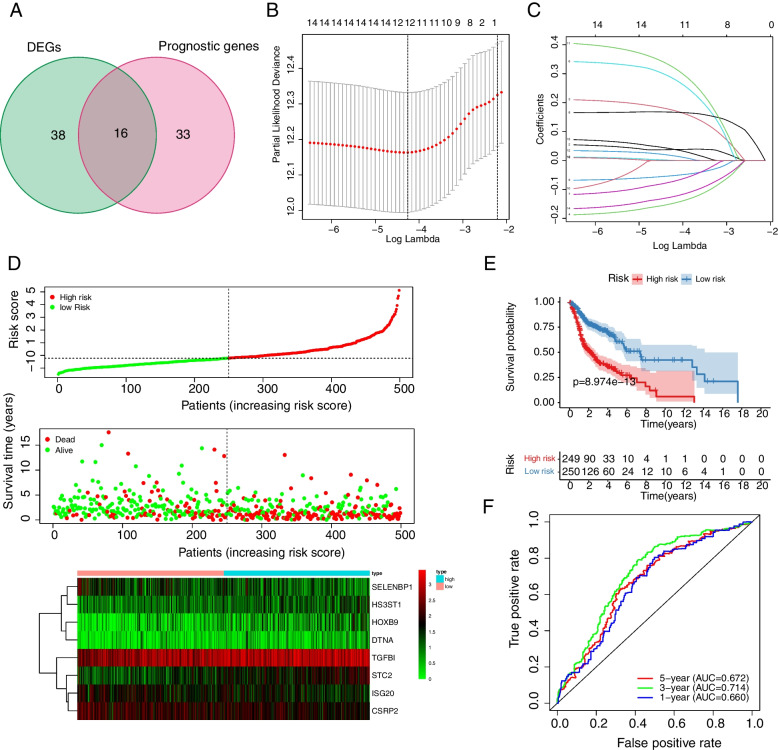
Development and evaluation of prognostic features based on hypoxia-associated genes in the TCGA database. **A** Venn diagram of differentially expressed prognostic-associated genes (DEGs is short for differentially expressed hypoxia-associated genes). **B** Five-fold cross-validation to select the optimal tuning parameter (λ). The right dashed line shows the optimal values by 1-SE criteria (λ = 0.0155201). **C** LASSO coefficient profiles of the 16 genes. Different color curves signify different genes. The numbers on the top of the figure indicate the number of the candidate genes entering in LASSO regression according to various lambda (λ) values displayed at the bottom of the figure. **D** Analysis of characteristic risk scores in the TCGA database. **E** Kaplan–Meier curves for low and high risk subgroups based on risk scores. **F** ROC curves for prognostic characteristics of patients with HNSCC

A univariate Cox regression approach was applied to screen HAGs significantly associated with OS in HNSCC. The analysis showed that 49 of 200 HAGs were highly related to OS in HNSCC, and 16 of these 49 genes were significantly different between HNSCC tissues and adjacent paracancerous tissues (Fig. 2A). To further narrow down the gene selection, LASSO analysis was performed on the 16 genes screened in the previous step and selected 12 genes [ Serpin family E member 1 (SERPINE1), Homeobox B9 (HOXB9), Selenium-binding protein 1 (SELENBP1), C-X-C motif chemokine receptor 4 (CXCR4), Dystrobrevin Alpha (DTNA), Interferon-stimulated exonuclease gene 20 (ISG20), Stanniocalcin 2 (STC2), Heparan Sulfate-Glucosamine 3-Sulfotransferase 1 (HS3ST1), Adrenomedullin (ADM), Stanniocalcin 1 (STC1), Cysteine and glycine-rich protein 2 (CSRP2), and Transforming growth factor beta-induced protein (TGFBI)] as candidate genes (Fig. 2B-C). Next, multifactorial Cox regression analysis was performed on the 12 candidate genes. Finally, eight genes (HOXB9, SELENBP1, DTNA, ISG20, STC2, HS3ST1, CSRP2, and TGFBI) were screened for the construction of prognostic risk models; among them, SELENBP1, CSRP2, and ISG20 were considered as biomarkers indicative of good prognosis (HR < 1), while TGFBI, STC2, HOXB9, DTNA, and HS3ST1 were indicative of poor prognosis (HR > 1). A risk model of HNSCC based on eight prognostic genes was generated (risk score = (0.199 × HOXB9 exp.) + (-0.238 × SELENBP1 exp.) + (0.359 × DTNA exp.) + (-0.134 × ISG20 exp.) + (0.193 × STC2 exp.) + (0.418 × HS3ST1 exp.) + (-0.159 × CSRP2 exp.) + (0.094 × TGFBI exp.)). Based on this risk model, the Kaplan–Meier method was applied to further investigate patients’ survival in HNSCC. 499 patients with HNSCC were distributed equally into two subgroups based on median risk scores, compared with the high-risk subgroup, those in the low-risk subgroup lived longer (Fig. 2D-E). Then, the model’s capability of predicting future HNSCC risk was evaluated using a time-dependent ROC analysis. The risk model predicted 1-year, 3-year, and 5-year survival with AUC values of 0.660, 0.714, and 0.672, respectively (Fig. 2F).

### Validation and clinical value of prognostic features

**Fig. 3:**
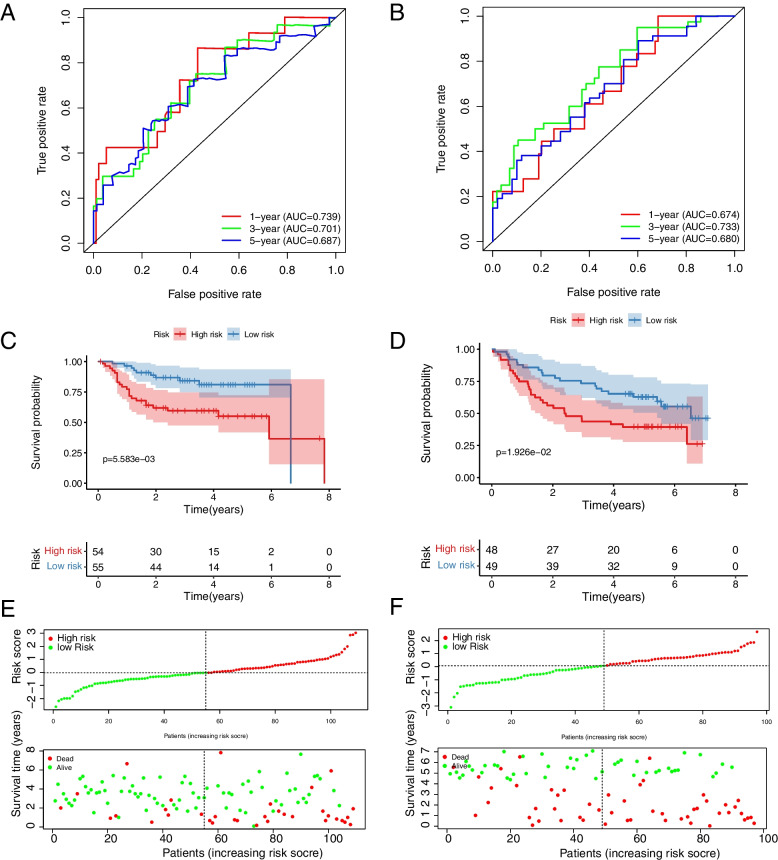
Prognostic analysis of the 8 hypoxia-associated gene signature in the datasets GSE27020 and GSE41613. **A** Time-dependent ROC analysis of HNSCC patients in the GSE27020 dataset. **B** Time-dependent ROC analysis of HNSCC patients in the GSE41613 dataset. **C** Kaplan–Meier survival analysis of HNSCC patients in the GSE27020 dataset. **D** Kaplan–Meier survival analysis of HNSCC patients in the GSE41613 dataset. **E** Characteristic risk score analysis of HNSCC patients in the GSE27020 dataset. **F** Characteristic risk score analysis of HNSCC patients in the GSE41613 dataset

The 8-gene prognostic risk model was validated in four GEO datasets (GSE27020, GSE41613, GSE42743, and GSE117973). According to the same risk score construction method in TCGA database, the four GEO datasets were classified into high and low risk groups based on the median risk scores and evaluated the performance of the risk model. The results of the analysis are shown in Figs. 3 and Additional file 3: Figure S1. The AUC for 1-year, 3-year, and 5-year OS was calculated to present the accuracy of the model prediction. In GSE27020 dataset, the AUC was 0.739, 0.701, and 0.687 respectively (Fig. 3A); In GSE41613 dataset, the AUC was 0.674, 0.733, and 0.680 respectively (Fig. 3B); In GSE42743 dataset, the AUC was 0.763, 0.717, and 0.789 respectively (Additional file 3: Figure S1A); In GSE117973 dataset, the AUC was 0.663, 0.748, and 0.736 respectively (Additional file 3: Figure S1B). Meanwhile, in all four GEO datasets (GSE27020, GSE41613, GSE42743, and GSE117973), patients in the low-risk group survived longer than those in the high-risk group (Fig. 3C-F, Additional file 3: Figure S1C-F). Taken together, the AUC of the risk score model for predicting the prognosis of patients in 3 years reached more than 0.7, indicating that the risk score model had a good value in predicting the prognosis of patients in three years.

### Association between the prognostic model and clinicopathological characteristics

Additionally, to explore the model’s prognostic value in HNSCC patients after stratification based on clinicopathological variables in the TCGA database, patients were classified into subgroups according to their Age, Gender, Grade, M stage, N stage, Radiation therapy, stage, and T stage to plot Kaplan–Meier survival curves. The sample was divided into 16 subgroups: Young (< 60 years) and Old (≥ 60 years), Female and Male, Grade I-II and Grade III-IV, Mstage M0 and Mstage M1 + Mx, Nstage N0 and Nstage N + , Radiation therapy NO and Radiation therapy YES, Stage I-II and Stage III-IV, Tstage T0-T2, and Tstage T3-T4. In each subgroup, the previous thresholds were selected and the patients were further allocated into the low and high groups. OS was significantly shorter in the high-risk group compared to the low-risk group across all subgroups (Additional file 3: Figure S2A-H, Additional file 3: Figure S3A-H). The results showed that the risk model could accurately predict the prognosis of HNSCC patients within the same clinicopathological subgroup. In conclusion, risk characteristics are crucial in determining a patient's prognosis for HNSCC.

### Comparison of different immune status between the two risk score-related subgroups

**Fig. 4:**
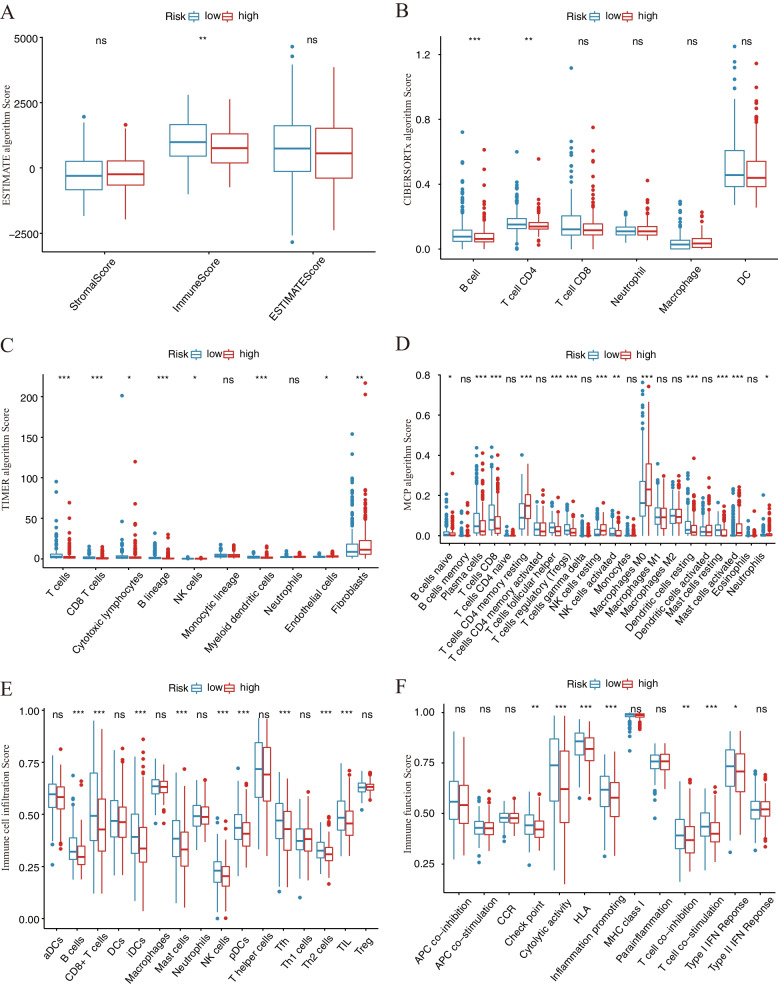
Comparison of immune analysis between different risk groups. **A** ESTIMATE algorithm. **B** CIBERSORTx algorithm. **C** TIMER algorithm. **D** MCP algorithm. **E** immune cell infiltration score by ssGSEA. **F** immune function score by ssGSEA. **A**-**F** compared the cellular components or cell immune responses between two groups. CCR, cytokine—cytokine receptor. Adjusted *P* values are shown as: ns, not significant; *P < 0.05; **P < 0.01; ***P < 0.001

To investigate the correlations between the risk scores model and immune status, immune cell infiltration and immune-related function scores were quantified by various algorithms (ESTIMATE, CIBERSORTx, TIMER, MCP counter, and ssGSEA). Figure 4 A demonstrates that the low-risk group's Immune Scores were significantly higher than those of the high-risk group, while the ESTIMATE Score and Stromal Score did not differ significantly, indicating a higher level of immunity in the low-risk group (*p* < 0.01). The variation of tumor microenvironment cells may be the cause of risk score heterogeneity. Then, based on TCGA-HNSCC data, analyses of the difference in immune cell subpopulations revealed significant differences between the two subgroups for scores of immune cells (B cells, T cells, Myeloid dendritic cells, plasma cells, Macrophage M0, T cell CD4 memory resting, T cell follicular helper, Tregs, NK cells resting, dendritic cells resting, Mast cells, iDCs, pDCs, Th2 cells, and TIL) (*p* < 0.001; Fig. 4B-E). Furthermore, immune function scores (Checkpoint, Cytolytic activity, HLA, Inflammation promoting, T cell co-inhibition, T cell co-stimulation, and Type I IFN response) were significantly different (*p* < 0.05; Fig. 4F). These results indicate that the types of immune infiltrating cells are different in the high and low risk groups, and the difference in immune microenvironment may be an important factor affecting the difference in the prognosis of patients.

### Construction of the Prognostic nomogram containing characteristic risk scores and clinical staging

**Fig. 5:**
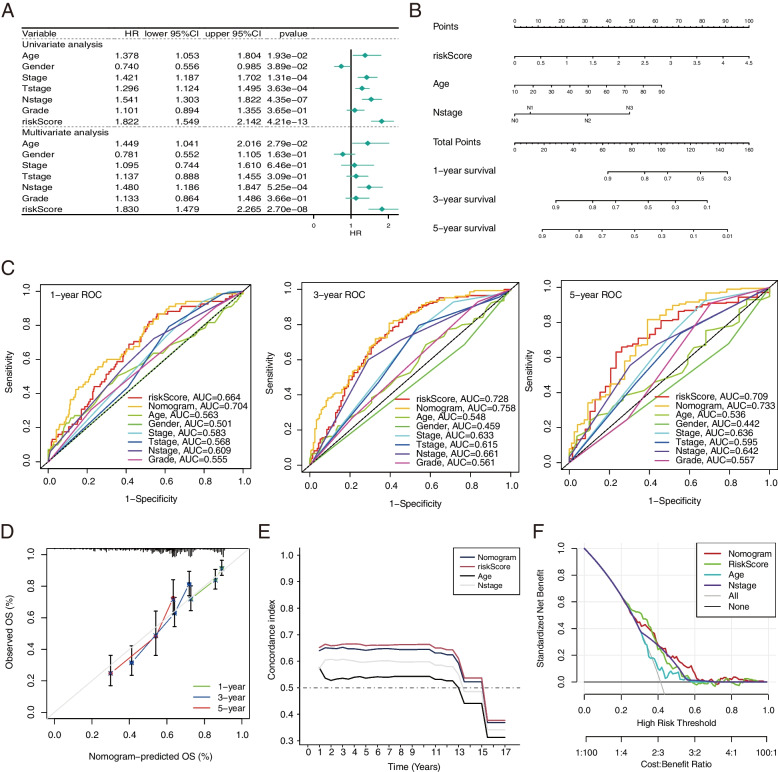
Nomogram predicting 1-, 3- and 5-year OS in the TCGA-HNSCC training dataset. **A** Univariable and multivariable analyses of the 8-gene signature in TCGA-HNSCC patients. **B** The development of a nomogram based on the 8-gene signature in the TCGA cohort for OS prediction at 1-, 3- and 5-year. **C** ROC analysis of 1-, 3- and 5-year OS prediction. **D** Calibration curves for prediction of 1-, 3- and 5-year OS. **E** Concordance index revealing measure of concordance of the predictor with patient survival. **F** DCA curves of the nomogram, riskscore, age and Nstage showed that the nomogram had good predictive performance

In univariate Cox regression analyses, risk score (HR = 1.822, 95% CI = 1.549–2.142, *p*-value < 0.001), Age (HR = 1.378, 95% CI = 1.053–1.804, *p*-value = 0.0193), Stage (HR = 1.421, 95% CI = 1.187–1.702, *p*-value < 0.001), Tstage (HR = 1.296, 95% CI = 1.124–1.495, *p*-value < 0.001), and Nstage (HR = 1.541, 95% CI = 1.303–1.822, p-value < 0.001) were correlated to OS in patients with HNSCC (Fig. 5A). In multivariate Cox regression analysis, characteristic risk score (HR = 1.830, 95% CI = 1.479–2.265, *p*-value < 0.001), Nstage (HR = 1.480, 95% CI = 1.186–1.847, *p*-value < 0.001) and Age (HR = 1.449, 95% CI = 1.041–2.016, *p*-value = 0.0279) were shown to be independent predictors of OS in HNSCC patients (Fig. 5A). Then, the risk score, Age, and N stage were combined to construct a nomogram for predicting OS of 1-year, 3-year, and 5-year in HNSCC patients (Fig. 5B). The performance of the nomogram was evaluated, and it showed superior predictive potential compared to the risk score and other clinical characteristics (Age, Gender, Stage, T stage, Nstage, and Grade) for OS at 1, 3, and 5 years (AUC = 0.704, 0.758 and 0.733 respectively, Fig. 5C). The calibration curves for the nomogram demonstrated good agreement between the actual OS probabilities and the OS probabilities predicted by the nomogram (Fig. 5D). The nomogram had good discrimination compared to the risk score, Age, and Nstage, with a C-index close to 0.7 (Fig. 5E). The DCA curves showed that the nomogram had more net benefit than the risk score, Age, and Nstage (Fig. 5F).

### Enrichment analysis based on hypoxia 8-gene signature

**Fig. 6:**
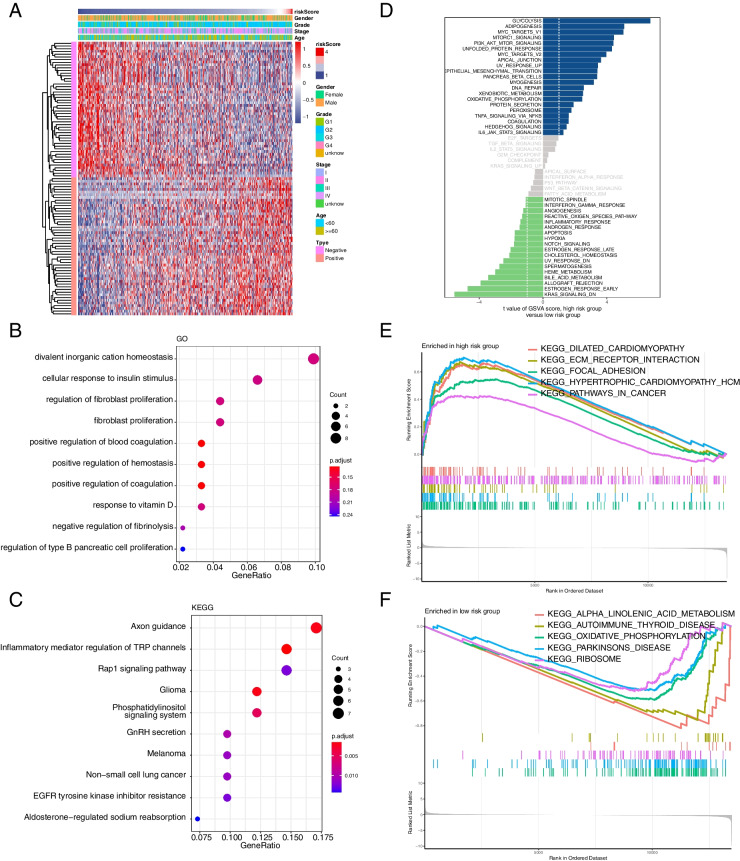
Functional enrichment analysis of risk score-associated genes. **A** Heatmap of the genes most associated with the hypoxia-associated gene signature (Pearson |R|> 0.5, *P* < 0.05). **B** GO enrichment analysis of the identified genes. **C** KEGG enrichment analysis of the identified genes. **D** GSVA enrichment analysis of the high and low risk groups. (E–F) GSEA enrichment analysis of the high and low risk groups

To clarify the signaling pathways related to the hypoxia 8-gene signature, we screened the genes with the highest correlation coefficients with risk scores (Pearson |R|> 0.5, *P* < 0.05) in the TCGA database and applied functional enrichment analysis to them. A total of 50 negatively and 51 positively correlated genes were screened by correlation analysis, and the correlation heatmap was presented in Fig. 6A. Next, GO enrichment analysis was performed on these 101 genes, which revealed that these genes were mainly involved in signaling pathways including divalent inorganic cation homeostasis, response to insulin stimulus, fibroblast proliferation, positive regulation of hemostasis and positive regulation of coagulation (Fig. 6B). Subsequently, KEGG enrichment analysis showed that they were involved in signaling pathways including Axon guidance, Inflammatory mediator regulation of TRP channels, Rap1 signaling pathway, and EGFR tyrosine kinase inhibitor resistance (Fig. 6C). Additionally, the signaling pathways involved in the hypoxia 8-gene signature were investigated using GSVAs and the results showed that in the high-risk subgroup, the top 5 signaling pathways significantly activated included Glycolysis, Adipogenesis, MYC Targets V1, MTORC1 Signaling, and PI3K-AKT-mTOR Signaling (Fig. 6D). Finally, the signaling pathways involved in the hypoxia 8-gene signature were screened by GSEA enrichment analysis, which showed that the significantly enriched signaling pathways in the high-risk subgroup included: Dilated cardiomyopathy, ECM Receptor interaction, Focal adhesion, Hypertrophic cardiomyopathy and pathways in cancer (Fig. 6E). Significantly enriched signaling pathways in the low-risk subgroup included Alpha-linolenic acid metabolism, Autoimmune thyroid disease, Oxidative phosphorylation, Parkinson disease, and Ribosome (Fig. 6F).

### Core gene expression validation

**Fig. 7:**
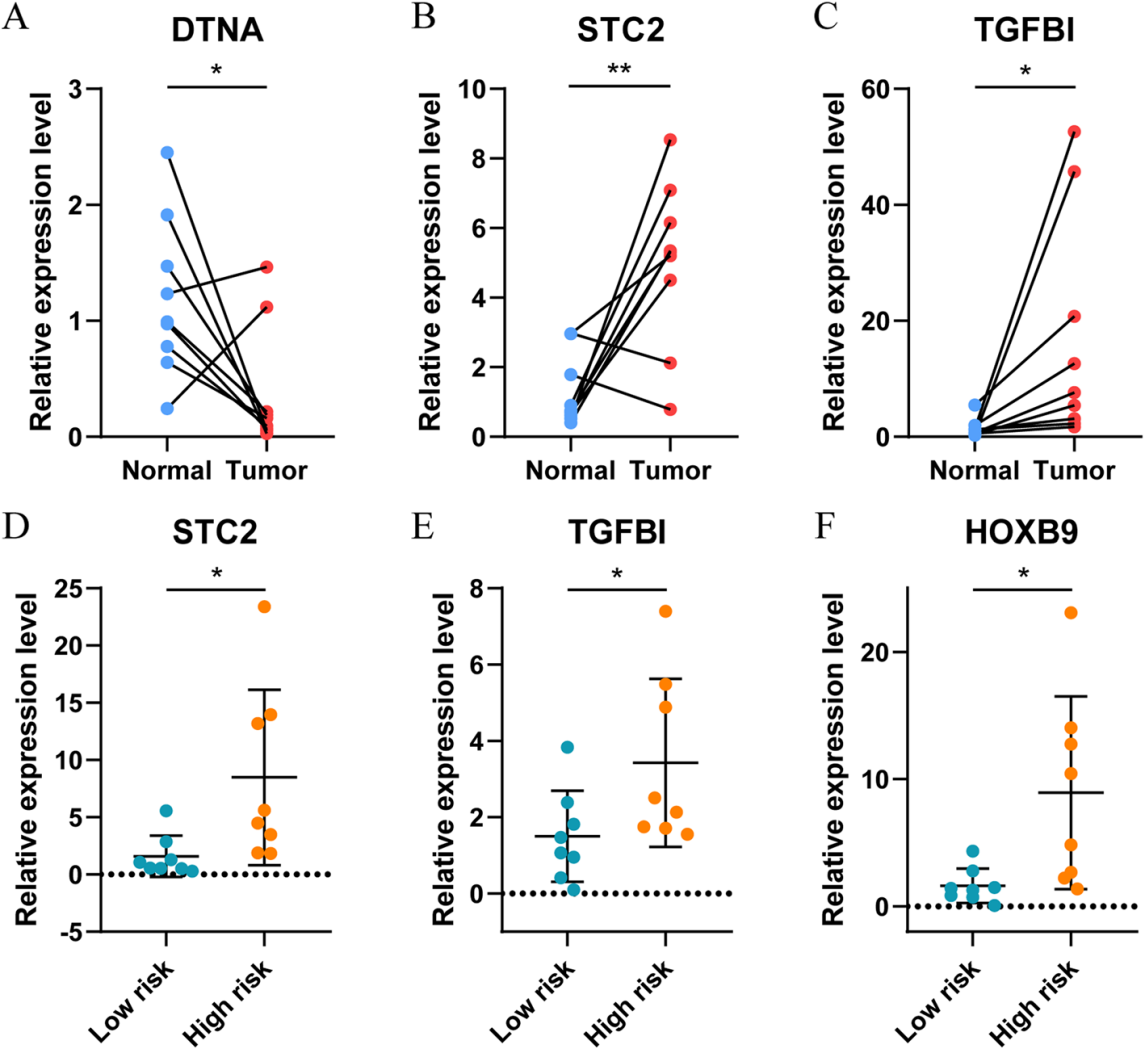
Validation of expression of core genes by quantitative real-time PCR (qRT-PCR). The figures of qRT-PCR showed the expression levels of DTNA (**A**), STC2 (**B**), and TGFBI (**C**) in adjacent normal tissues and HNSCC tissues (*n* = 9, each group); the figures of qRT-PCR showed the expression levels of STC2 (**D**), TGFBI (**E**), and HOXB9 (**F**) in low and high risk groups (*n* = 8, each group).*P < 0.05, **P < 0.01, ***P < 0.001

To determine the expression of 8 hypoxia-related genes in HNSCC tumor tissues, we collected 9 pairs of tissues from oral squamous cell carcinoma patients for qRT-PCR verification. There was no difference in HOXB9, SELENBP1, ISG20, HS3ST1 and CSRP2 expression between tumor and normal tissues (Additional file 3: Figure S4A-E). Three genes (DTNA, STC2, and TGFBI) were differentially expressed between HNSCC tumor tissue and normal tissue, and STC2 and TGFBI were significantly up-regulated in tumor tissue, which was consistent with the analysis results (Fig. 7A-C). Furthermore, the risk model was used to divide 16 HNSCC patients into high and low risk groups. qPCR detection found that STC2, TGFBI and HOXB9 were significantly up-regulated between the high and low risk group (Fig. 7D-F), while SELENBP1, DTNA, ISG20, HS3ST1 and CSRP2 had no significant differences (Additional file 3: Figure S4F-J).

## Discussion

HNSCC is characterized by late diagnosis, easy metastasis, relapse, and resistance to treatments. The five-year survival rate of patients is very low, which seriously endangers the health of patients [24]. Although great progress has been made in diagnosis and treatment strategies in the past decades, the overall survival rate of HNSCC patients has not been significantly improved [25]. Therefore, it is necessary to explore new prognostic prediction schemes to accurately assess the tumor progression and survival status of patients. Hypoxia is a common phenomenon in tumor tissues, which has a wide impact on tumor angiogenesis, proliferation, migration, and the prognosis of cancer patients [26, 27]. So far, hypoxia-associated genes have been used as risk factors to establish prognostic risk models for a variety of tumors, including liver cancer, breast cancer, bladder cancer and so on [28–30]. In HNSCC, the effect of hypoxia on tumor progression has been demonstrated. However, the exact molecular mechanism of hypoxia-associated genes in HNSCC is still unclear, and their prognostic value is far from elucidated.

In this study, 54 differentially expressed hypoxia-associated genes in HNSCC patient samples from TCGA database were screened by differential expression analysis. Eight hypoxia-associated genes (SELENBP1, CSRP2, ISG20, TGFBI, STC2, HOXB9, DTNA and HS3ST1) with high predictive value were screened by univariate Cox regression analysis, LASSO regression analysis and multivariate Cox regression analysis. Finally, a prognostic risk model for HNSCC was constructed. The prognostic value of the risk model was validated in four HNSCC datasets (GSE27020, GSE41613, GSE42743 and GSE117973) from the GEO database. Kaplan–Meier survival analysis and ROC curve analysis showed that the AUC values of 5-year survival predicted by this model were 0.672 (TCGA), 0.687 (GSE27020), 0.680 (GSE41613), 0.789 (GSE42743) and 0.736 (GSE117973), significantly better than previously reported HNSCC prognostic models (AUC = 0.607) [31]. The prognosis prediction model we constructed contains 8 hypoxia-associated genes, which is significantly better than the 24-gene prognosis model constructed by Ding et al. [13]. Reduction in the number of genes reduced the difficulty of detection. In addition, Ding et al. only compared the prognostic value of risk scores and clinicopathological factors as independent prognostic factors. Our study not only assessed the predictive value of risk scores and clinicopathological factors as independent prognostic factors, but also combined risk scores with independent prognostic factors such as Age and N stage to build a nomogram. Most importantly, compared to Ding et al., we used clinical samples to validate differences in predictive genes between tumors and adjacent normal tissues and between high-low risk groups.

The eight hypoxia-associated genes identified in this study have been studied in the field of tumor. As a member of the family of selenium-binding proteins, SELENBP1 is a tumor suppressor, and its low expression has been reported to contribute to a poor prognosis in the lung [32], ovarian [33], and colorectal cancers [34]. CSRP2 is involved in tumor cell proliferation, migration, and invasion in breast cancer [35], gastric cancer [36], and lymphocytic leukemia [37]. CSRP2 is associated with a better prognosis in oral squamous cell carcinoma [38]. ISG20 is a 3'-5' exonuclease that can degrade viral RNA in vitro [39]. In human gliomas, patients expressing high ISG20 had a poor prognosis, which was inconsistent with our study [40]. The inconsistent may be due to cancer type specific that gliomas are a non-epithelial cell derived whereas HNSCC is epithelial cell malignancy. These studies revealed the tumor suppressor role of SELENBP1, CSRP2, and ISG20, whose low expression is often a risk factor for poor tumor prognosis. In this study, we found that low expression of SELENBP1, CSRP2 and ISG20 in HNSCC has predictive value for poor prognosis of HNSCC. Therefore, SELENBP1, CSRP2 and ISG20 were included as part of the influencing factors to construct a prognostic prediction model in this study.

In addition, TGFBI is a secreted extracellular matrix (ECM) protein that is induced by transforming growth factor β (TGFβ). It is reported that p-EMT-related genes including TGFBI were highly expressed in HNSCC samples compared to normal tissue, and this was linked to a poor prognosis [41]. Hypoxia-induced EMT has been demonstrated in a variety of tumors, and whether upregulated TGFBI under hypoxia affects the prognosis of HNSCC patients through EMT requires follow-up studies. Under hypoxia conditions, the expression of glycoprotein hormone STC2 is activated [42], which drives the growth, proliferation, and tumorigenesis of tumor cells. HOXB9 is a HOX gene involved in the regulation of several human cancers [43]. DTNA encodes a scaffolding protein that keeps muscle cells structurally intact. Previous study have identified DTNA as a valuable diagnostic marker for colon adenocarcinoma [44]. In the analysis results, DTNA was highly expressed in tumor tissues, and in the validation results, DTNA expression was downregulated in tumor samples; this inconsistency may be due to the selection of advanced HNSCC tissues for validation. HS3ST1 is a rate-limiting enzyme involved in the biosynthesis of heparan sulfate. Conditional deletion of HS3ST1 significantly inhibited tumor development in colorectal cancer [45]. These studies suggest tumor promotion by TGFBI, STC2, HOXB9, DTNA, and HS3ST1. In our study, high expression of TGFBI, STC2, HOXB9, DTNA and HS3ST1 was found to be associated with poor prognosis in HNSCC. Whether these genes contribute to the progression of HNSCC by a similar mechanism requires further investigation.

It is reasonable to infer that the eight genes we identified as a whole had high prognostic value for HNSCC. Additionally, we combined characteristic risk scores and clinical staging to develop a nomogram. The nomogram calibration curves predicted the OS of HNSCC patients more accurately. Meanwhile, we used ESTIMATE, TIMER, MCP counter, CIBERSORTx, and single sample gene set enrichment analysis (ssGSEA) to assess the immune state between various risk groups. The results showed differences in immune cell scores between the high and low risk groups, suggesting that risk scores may influence the prognosis of HNSCC patients through tumor microenvironment. Finally, qRT-PCR was performed on eight genes (HOXB9, SELENBP1, DTNA, ISG20, STC2, HS3ST1, CSRP2, and TGFBI). According to risk score, HNSCC samples were grouped into high and low risk groups. STC2, TGFBI and HOXB9 were found significantly up-regulated in the high-risk group.

In recent years, several studies[46–48]have been dedicated to establishing prognostic signatures related to hypoxia in HNSCC patients using the TCGA database. However, our study employed distinct approaches, such as multi-dataset validation, multi-algorithm for immune infiltration analysis, combined clinicopathological features of patients, clinical sample validation, etc.

This study is based on database data mining and preliminary qPCR experimental validation, which still has some limitations. Firstly, the limited sample size may lead to selection bias. To make the results more stable, more clinical cases should be further included to carry out large sample studies. Secondly, this study verified the expression trend of 8 HAGs at transcript level between tumor and normal tissues, as well as between high and low risk groups based on qPCR. Whether there is a corresponding trend at the protein level needs further study. In addition, for better clinical application value, large-sample multi-center clinical trials and prospective studies are still needed to confirm the prognostic predictive value of the diagnostic model. Finally, functional and mechanism studies of the identified hypoxia-related genes in HNSCC may also be new directions.

## Conclusions

We have developed a risk model based on 8 HAGs to predict prognosis in HNSCC patients. Additionally, we developed a nomogram which showed better predictive performance than the risk score and clinical characteristics. Our findings suggest that the HAGs-based risk model has important value in prognostic prediction and clinical decision making in HNSCC.

### Supplementary Information

**Additional file 1: Table S1.** Clinical information of patients with HNSCC included in this study. **Table S2.** Baseline information of the included clinical samples. **Table S3. **The primer sequence information of qPCR experiment.

**Additional file 2.** Details of patients in the TCGA database.

**Additional file 3: Figure S1.** Prognostic analysis of the 8 hypoxia-associated gene signature in the datasets GSE42743 and GSE117973. (A) Time-dependent ROC analysis of HNSCC patients in the GSE42743 dataset. (B) Time-dependent ROC analysis of HNSCC patients in the GSE117973 dataset. (C) Kaplan-Meier survival analysis of HNSCC patients in the GSE42743 dataset. (D) Kaplan-Meier survival analysis of HNSCC patients in the GSE117973 dataset. (E) Characteristic risk score analysis of HNSCC patients in the GSE42743 dataset. (F) Characteristic risk score analysis of HNSCC patients in the GSE117973 dataset. **Figure S2.** Kaplan-Meier survival curves for the high-risk and low-risk groups stratified by clinical characteristics. (A) Age ≥60 years, (B) Age < 60 years, (C) Gender (female), (D) Gender (male), (E) Grade I-II, (F) Grade III-IV, (G) Mstage M0 and (H) Mstage M1+Mx. **Figure S3.** Kaplan-Meier survival curves for the high-risk and low-risk groups stratified by clinical characteristics. (A) Nstage N+, (B) Nstage N0, (C) Radiation therapy NO, (D) Radiation therapy YES, (E) Stage I-II, (F) Stage III-IV, (G) Tstage T0-T2 and (H) Tstage T3-T4.

## Data Availability

The datasets presented in this study can be found in GEO (https://www.ncbi.nlm.nih.gov/) and TCGA (https://www.genome.gov/Funded-Programs-Projects/Cancer-Genome-Atlas) databases.

## Abbreviations

AUC: Area under the curve
CI: Confidence interval
C-index: Consistency index
DCA: Decision curve analysis
GEO: Gene Expression Omnibus
GSEA: Gene set enrichment analysis
GSVA: Gene set variation analysis
HAG: Hypoxia-associated gene
HNSCC: Head and neck squamous cell carcinoma
HR: Hazard ratio
OS: Overall survival
qRT-PCR: Quantitative real-time polymerase chain reaction
RNA-seq: RNA sequence
ROC: Receiver operating characteristic
ssGSEA: Single-sample gene set enrichment analysis
