# Supplementary material for: A novel hypoxia-associated gene signature for prognosis prediction in head and neck squamous cell carcinoma
Source: BMC Oral Health. 2023 Nov 14;23:864. doi: 10.1186/s12903-023-03489-8 (PMC10647095; doi:10.1186/s12903-023-03489-8)
Supplement: Supplementary file 1 — Additional file 1: Table S1. Clinical information of patients with HNSCC included in this study. Table S2. Baseline information of the included clinical samples. Table S3. The primer sequence information of qPCR experiment. [file 12903_2023_3489_MOESM1_ESM.docx]

**Table S1. Clinical information of patients with HNSCC included in this study**

|  | TCGA cohort | GSE27020 | GSE41613 | GSE42743 | GSE117973 |
| --- | --- | --- | --- | --- | --- |
| **No. of patients** | 499 | 109 | 97 | 74 | 77 |
| **Age (median, range)** | 61.5 (19-90) | 63.3 (41-88) | - | 58.12（22-84） | 60.89 (39.68-82.52) |
| **Gender (%)** |  |  |  |  |  |
| Female | 133 | - | 31 | 16 | 17 |
| Male | 366 | - | 66 | 58 | 60 |
| **Grade** |  |  |  |  |  |
| G1 | 61 | 42 | - | 3 | 1 |
| G2 | 298 | 49 | - | 15 | 46 |
| G3 | 119 | 16 | - | 15 | 30 |
| G4 | 2 | 0 | - | 40 | - |
| **HPV infection** |  |  |  |  |  |
| HPV-negative | 19 | - | 97 | - | 54 |
| HPV-positive | 41 | - | 0 | - | 23 |
| **Survival status** |  |  |  |  |  |
| Dead | 216 | 75 | 46 | 42 | 22 |
| Alive | 283 | 34 | 51 | 32 | 55 |

**Table S2. Baseline information of the included clinical samples**

| Site | gender | Age | Grade | Stage | T | M | N |
| --- | --- | --- | --- | --- | --- | --- | --- |
| Cheek mucosa | male | 70 | G2 | Stage IVA | T3 | M0 | N2 |
| Floor of mouth | female | 43 | G2 | Stage IVA | T4a | M0 | N2c |
| Tongue | male | 51 | G1 | Stage IVC | T2 | M1 | N1 |
| Base of tongue | male | 62 | G3 | Stage IVB | T2 | M0 | N3 |
| Floor of mouth | male | 61 | G2 | Stage II | T2 | M0 | N0 |
| Cheek mucosa | male | 75 | G2 | Stage II | T2 | M0 | N0 |
| Tongue | female | 60 | G3 | Stage III | T3 | M0 | N1 |
| Tongue | male | 52 | G3 | Stage IVA | T4a | M0 | N0 |
| Tongue | male | 44 | G3 | Stage II | T2 | M0 | N0 |
| Cheek mucosa | male | 57 | G2 | Stage IVA | T4 | M0 | N0 |
| Tongue | male | 63 | G2 | Stage I | T1 | M0 | N0 |
| Cheek mucosa | male | 70 | G2 | Stage II | T2 | M0 | N0 |
| Floor of mouth | male | 53 | G2 | Stage IVA | T4a | M0 | N1 |
| Tongue | female | 80 | G2 | Stage II | T2 | M0 | N0 |
| Floor of mouth | male | 86 | G3 | Stage II | T2 | M0 | N0 |
| Tongue | male | 44 | G3 | Stage II | T2 | M0 | N0 |

**Table S3.** **The primer sequence information of qPCR experiment**

| Gene | Forward Sequence | Reverse Sequence |
| --- | --- | --- |
| HS3ST1 | GCGTGCTATCTGACTACACCCA | GCCTTGTAGTCCACATTGAGCC |
| DTNA | CGGCTTGATGAAGAACACAGGC | GATGTCAGGAGCACTTCTCTGC |
| HOXB9 | TGCGAAGGAAGCGAGGACAAAG | TCCTTCTCTAGCTCCAGCGTCT |
| STC2 | GCATGACTTTTCTGCACAACGCT | GGCTTATGCAGCCGAACCTGTG |
| TGFBI  ISG20  CSRP2  SELENBP1 | GGACATGCTCACTATCAACGGG  ACACGTCCACTGACAGGCTGTT  GAAGAGGTGCAGTGTGATGGCA  TTGGAGATCCGCTTCCTGCACA | CTGTGGACACATCAGACTCTGC  ATCTTCCACCGAGCTGTGTCCA  GCCCATACTTCTTTCCGTAGCAG  GGATCACCTTCTCCACTGACCA |
| GAPDH | GTCTCCTCTGACTTCAACAGCG | ACCACCCTGTTGCTGTAGCCAA |
