# Supplementary material for: A novel hypoxia-associated gene signature for prognosis prediction in head and neck squamous cell carcinoma
Source: BMC Oral Health. 2023 Nov 14;23:864. doi: 10.1186/s12903-023-03489-8 (PMC10647095; doi:10.1186/s12903-023-03489-8)

**Figure S1** **Prognostic analysis of the 8 hypoxia-associated gene signature in the GSE42743 and GSE117973.**

(A) Time-dependent ROC analysis of HNSCC patients in the GSE42743. (B) Time-dependent ROC analysis of HNSCC patients in the GSE117973. (C) Kaplan-Meier survival analysis of 103 HNSCC patients in the GSE42743. (D) Kaplan-Meier survival analysis of HNSCC patients in the GSE117973. (E) Characteristic risk score analysis of HNSCC patients in the GSE42743. (F) Characteristic risk score analysis of HNSCC patients in the GSE117973.


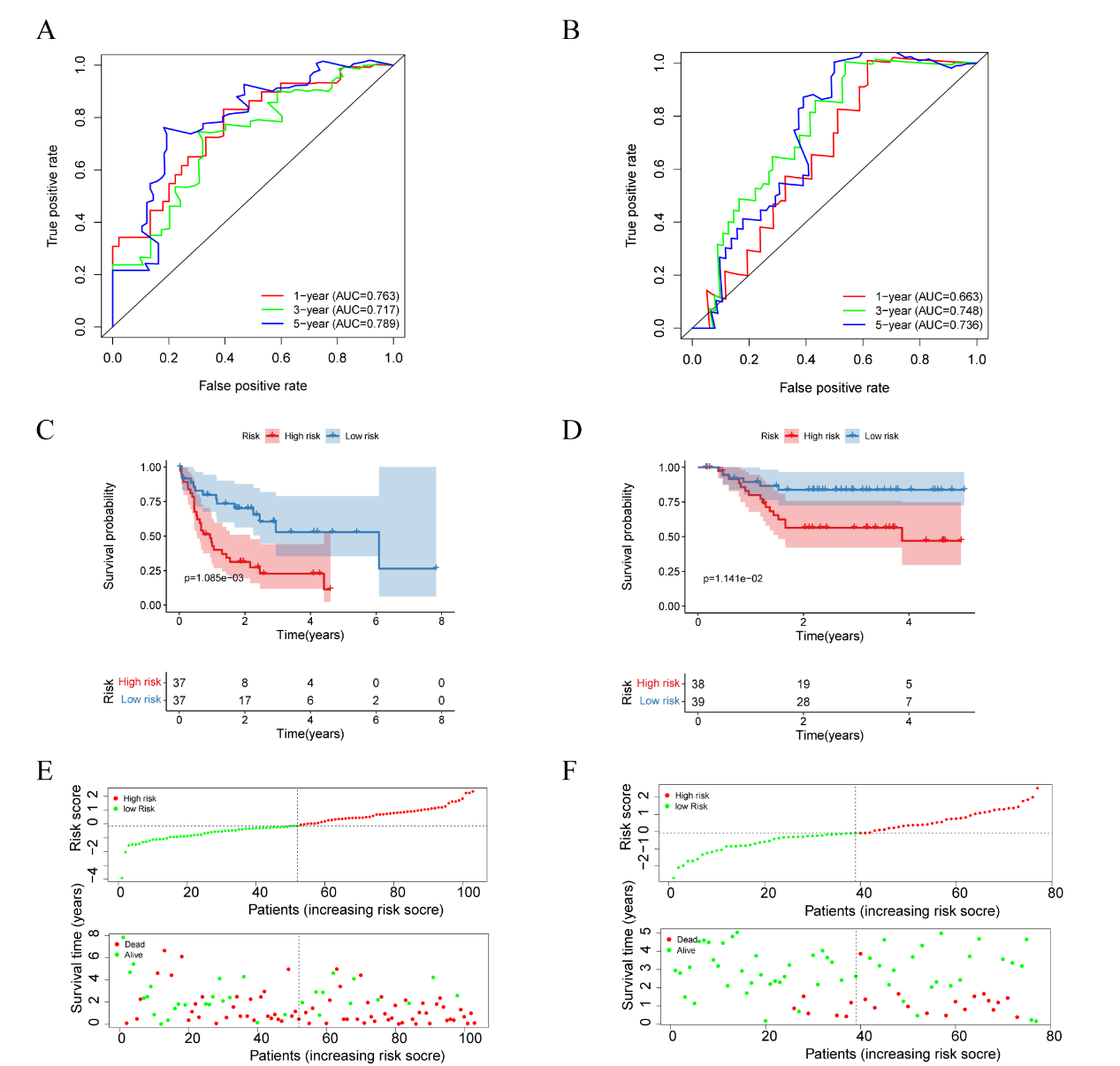


**Figure S2. Kaplan-Meier survival curves for the high-risk and low-risk groups stratified by clinical characteristics.**

(A) Age ≥60 years, (B) Age < 60 years, (C) Gender (female), (D) Gender (male), (E) Grade I-II, (F) Grade III-IV, (G) Mstage M0 and (H) Mstage M1+Mx.


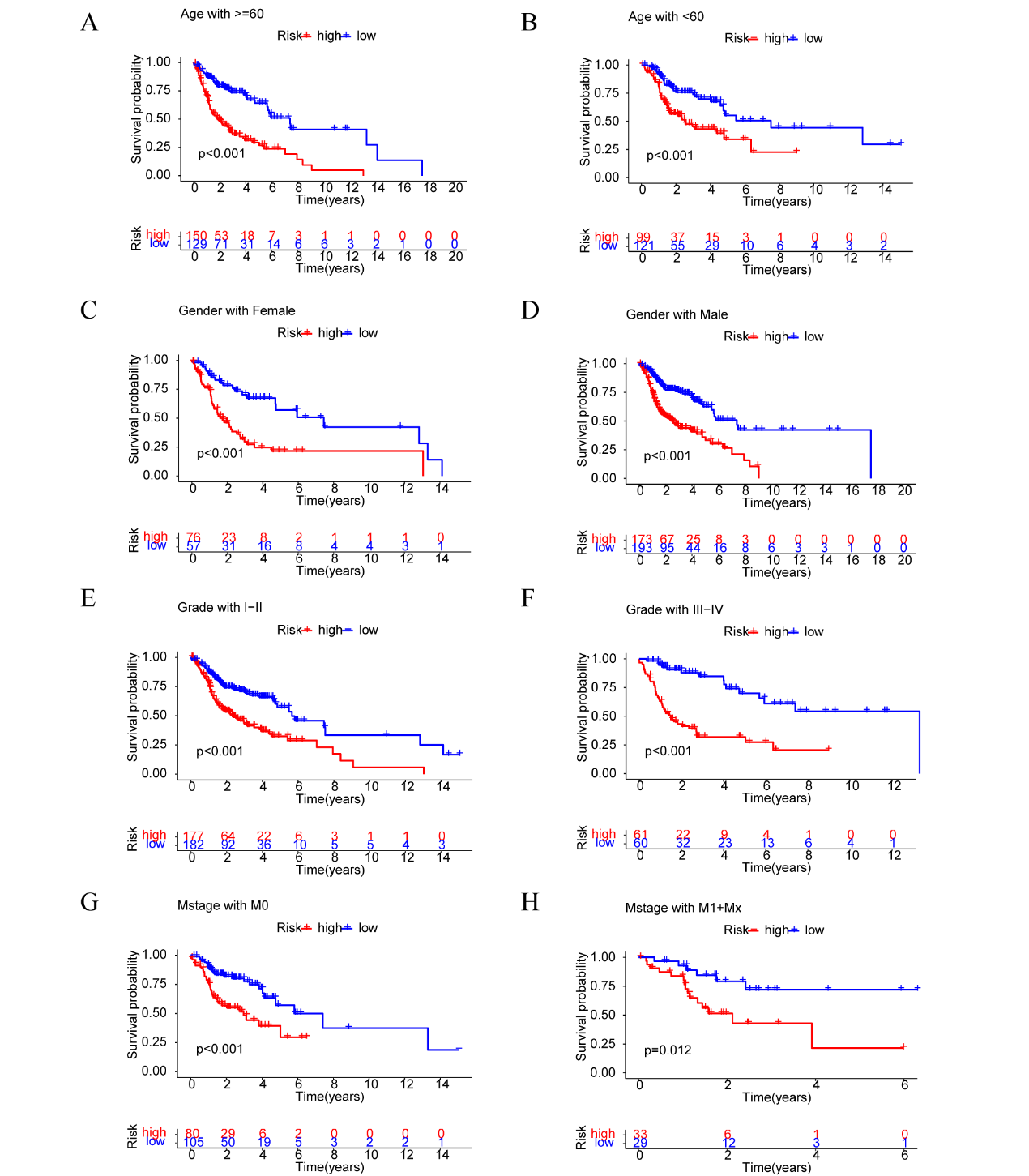


**Figure S3. Kaplan-Meier survival curves for the high-risk and low-risk groups stratified by clinical characteristics.**

(A) Nstage N+, (B) Nstage N0, (C) Radiation therapy NO, (D) Radiation therapy YES, (E) Stage I-II, (F) Stage III-IV, (G) Tstage T0-T2 and (H) Tstage T3-T4.


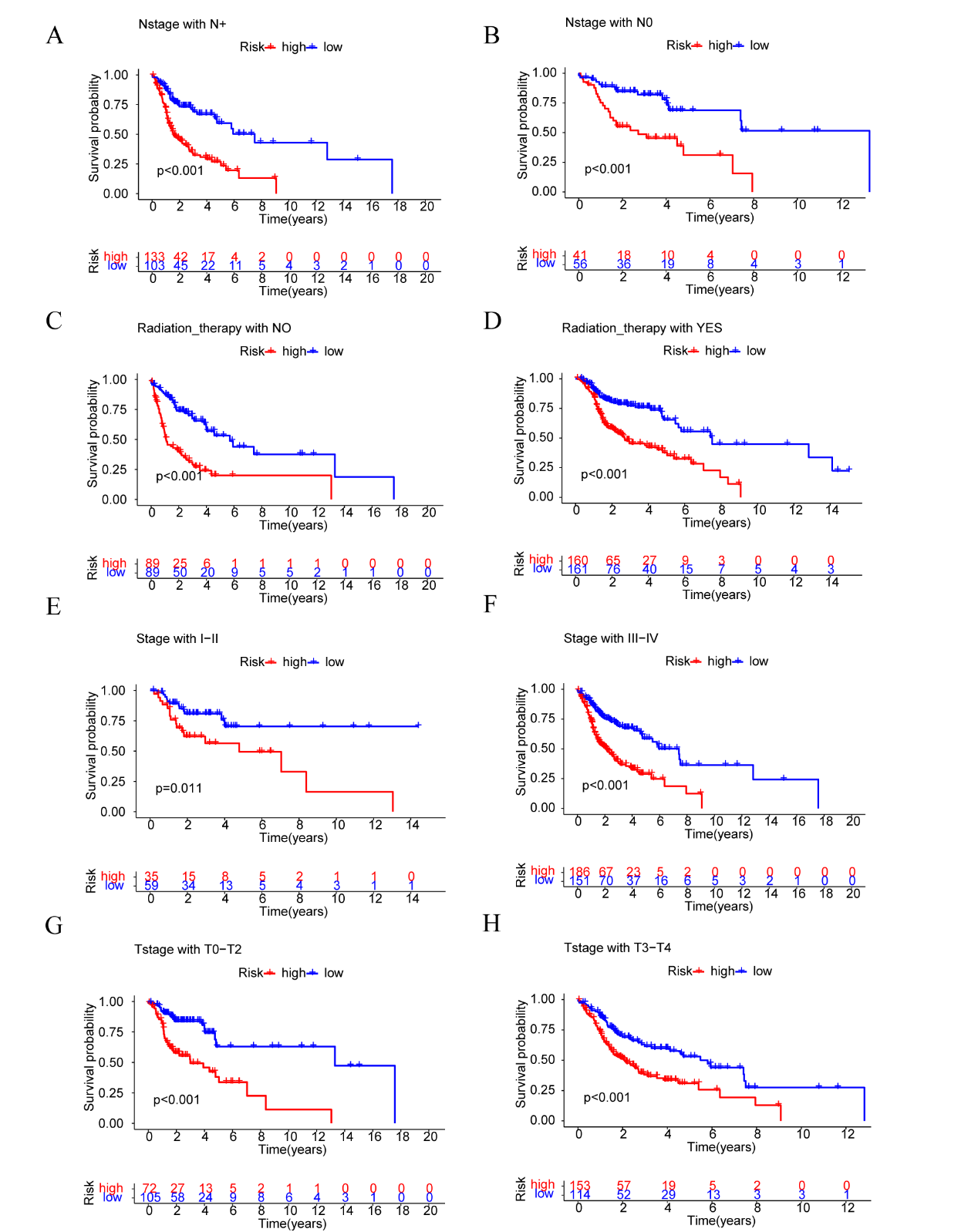


**Figure S4. Validation of expression of core genes by quantitative real-time PCR (qRT-PCR).**

The figures of qRT-PCR showed the expression levels of HOXB9 (A), SELENBP1(B), ISG20(C), HS3ST1 (D) and CSRP2(E) in adjacent normal tissues and HNSCC tissues (n=9, each group); the figures of qRT-PCR showed the expression levels of SELENBP1 (F), DTNA (G), ISG20(H), HS3ST1 (I) and CSRP2 (G) in low and high risk groups (n=8, each group). ns denotes no statistical difference.


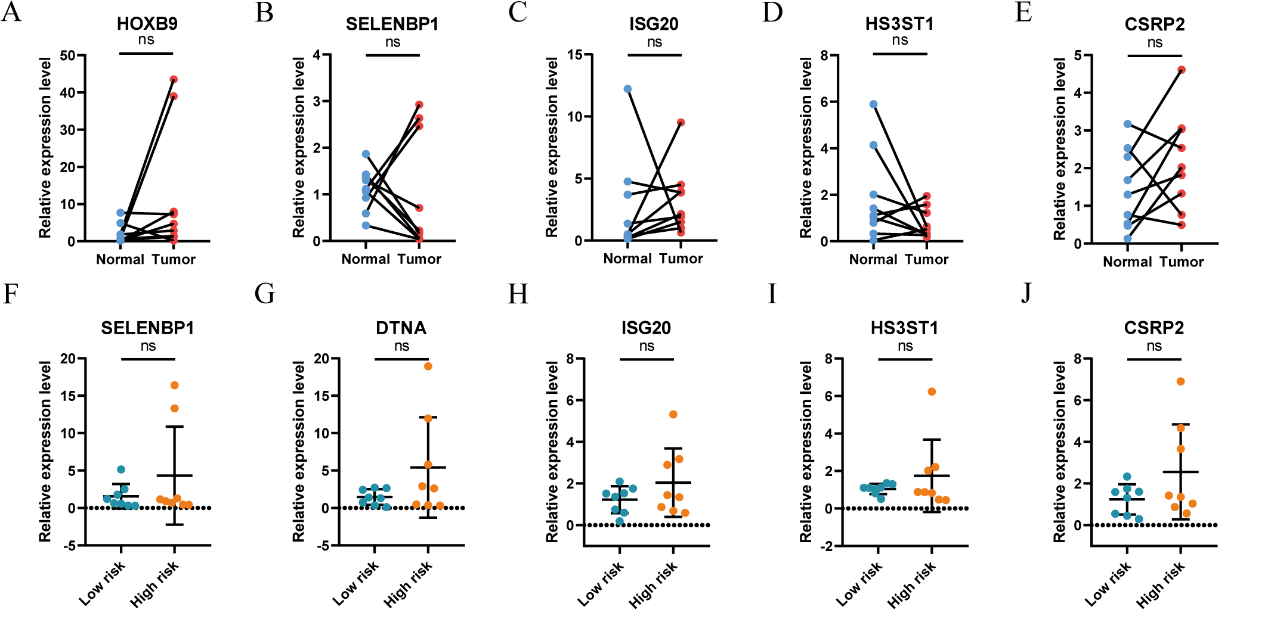

Supplement: Supplementary file 3 — Additional file 3: Figure S1. Prognostic analysis of the 8 hypoxia-associated gene signature in the datasets GSE42743 and GSE117973. (A) Time-dependent ROC analysis of HNSCC patients in the GSE42743 dataset. (B) Time-dependent ROC analysis of HNSCC patients in the GSE117973 dataset. (C) Kaplan-Meier survival analysis of HNSCC patients in the GSE42743 dataset. (D) Kaplan-Meier survival analysis of HNSCC patients in the GSE117973 dataset. (E) Characteristic risk score analysis of HNSCC patients in the GSE42743 dataset. (F) Characteristic risk score analysis of HNSCC patients in the GSE117973 dataset. Figure S2. Kaplan-Meier survival curves for the high-risk and low-risk groups stratified by clinical characteristics. (A) Age ≥60 years, (B) Age < 60 years, (C) Gender (female), (D) Gender (male), (E) Grade I-II, (F) Grade III-IV, (G) Mstage M0 and (H) Mstage M1+Mx. Figure S3. Kaplan-Meier survival curves for the high-risk and low-risk groups stratified by clinical characteristics. (A) Nstage N+, (B) Nstage N0, (C) Radiation therapy NO, (D) Radiation therapy YES, (E) Stage I-II, (F) Stage III-IV, (G) Tstage T0-T2 and (H) Tstage T3-T4. [file 12903_2023_3489_MOESM3_ESM.docx]
